# Prevalence and correlates of self-reported disordered eating: A cross-sectional study among 90 592 middle-aged Norwegian women

**DOI:** 10.1371/journal.pone.0211056

**Published:** 2019-01-23

**Authors:** Marie Sigstad Lande, Jan H. Rosenvinge, Guri Skeie, Charlotta Rylander

**Affiliations:** 1 Department of Community Medicine, UiT The Arctic University of Norway, Tromsø, Norway; 2 Department of Psychology, UiT The Arctic University of Norway, Tromsø, Norway; Leibniz Institute for Prevention Research and Epidemiology BIPS, GERMANY

## Abstract

Disordered eating (DE) is extensively studied among adolescents and young women. However, there is growing evidence that DE as well as the clinical eating disorders may occur at any age from childhood to advanced years. This study aimed to determine the prevalence and correlates of DE in a representative sample of middle-aged women from Norway. The study included 90 592 women (median age: 55 years) from the Norwegian Women and Cancer study who responded to a questionnaire between the years 2002–2005. Correlates of self-reported DE were assessed by logistic regression analyses. The overall period prevalence of DE between 2002–2005 was 0.28 (95% confidence interval 0.25–0.31) %, and was highest among women ≥ 66 years: 0.65 (0.60–0.70) %. DE was strongly associated with depression (Odds ratio [OR] 3.34 [95% confidence interval 2.53–4.41]), being unemployed (OR 1.78 [1.32–2.40]) and single (OR 1.66 [1.25–2.20]). Women with DE were more likely to report low energy intake (OR 1.41 [1.08–1.86]) and were less likely to be moderately physically active (OR 0.67 [0.47–0.95]). Using the largest study sample in the literature, the present findings confirm smaller studies showing that DE do occur in women in mid-life and older age as well. Our results contribute to address a somewhat under-communicated community health problem that needs attention in terms of age-specific treatment and prevention.

## Introduction

Body dissatisfaction, weight preoccupation, and dysfunctional eating patterns represent the core features of disordered eating (DE). DE, as well as the diagnostic counterparts, anorexia nervosa (AN), bulimia nervosa (BN) and binge eating disorder (BED) have been conceptualized as juvenile problems in terms of etiology (e.g., pre-pubertal risk factors and developmental age specific challenges) and peak prevalence [1, 2]. However, there is growing evidence that DE as well as the clinical eating disorders may occur at any age from childhood to advanced years. Since 1986, several reviews of late onset DE have shown that also developmental challenges related to life transition issues in midlife may elicit DE [3–6]. High prevalence of DE features among older women has also been reported: In a study of 536 women aged 50–64 years, 27% were concerned about being too fat [7] and among a random sample of 1000 women aged 60–70 years, 60% reported body dissatisfaction [8]. Ng et al. reported a prevalence of 2.6% of DE among 2870 women above 50 years [9]. Another study by Runfola et al. found that body dissatisfaction was highly prevalent (72–93%) among adult women aged 35 to 75 and above, and with no age difference [10]. The latter study contrast other large (n = 1800–27 252) population studies [2, 11–13], which found an inverse relationship between age and prevalence of eating disorders/DE. For instance, a population-based study of about 25 000 Norwegians from mid-Norway reported an overall prevalence of 12% of DE among women above 30 years in 2006–2008 and a negative association with age [13]. Previous studies have also suggested that the focus on thin female body ideals and concerns about being too fat seem to level off after the age of 60 [7, 14]. Thus, although DE is likely more prevalent among younger women, it is still a challenge for women in advanced years and often associated with serious comorbidities. DE in older adults has for instance been associated with higher BMI, and about six times increased risk of anxiety, mood disorders and alcohol dependence [7, 15–20].

Across studies, DE is one of the most consistent risk factors for AN, BN, and BED [21]. Considering the fact that incident cases of BN and BED may emerge at ages above 50 [2], a monitoring of DE among adults in the general population is highly warranted in order to scale treatment and prevention efforts. Additionally, there is currently little information about the prevalence of DE among the general Norwegian female population above 40 years of age. Thus, the aim of the present study was to estimate the prevalence of DE in a representative sample of 90 592 Norwegian women and to explore sociodemographic and lifestyle-related correlates.

## Methods

### Study design and participants

The Norwegian Women and Cancer Study (NOWAC) is a national representative cohort study that was initiated in 1991, aiming to explore the associations between lifestyle factors and cancer among Norwegian women [22]. A random selection of women between the age of 30 and 70 years from the National Registry of Norway were invited to participate in the study through a letter with a detailed questionnaire sent to their home address. Those who agreed to participate have been followed up regularly with consecutive questionnaires every 6-7^th^ year. The cohort has been expanded several times, and at present, it includes more than 170 000 Norwegian women and encompasses several other health outcomes than cancer. The NOWAC questionnaire includes questions about sociodemographic variables, self-reported health and lifestyle habits including use of various medications and a detailed food frequency questionnaire. The questionnaires have been distributed in waves, and some variables have been common in all waves, while other variables have varied depending on age groups and hypotheses. The external validity of the NOWAC study has been found to be satisfactory, with no major source of selection bias [23]. The NOWAC study has been approved by the Regional Committee for Medical Research Ethics in Northern Norway and the Norwegian Data Inspectorate. All participants gave their written informed consent.

The present study includes 90 592 women who answered a NOWAC questionnaire between the years 2002–2005 where a question related to eating disorders were included. Some women were first responders while others had answered at least one NOWAC questionnaire before. The response rate in NOWAC has varied between 48% (first responders) to 81% (second responders) [22]. No exclusion criteria were applied to the initial sample.

### DE and covariates

Information about self-reported DE was extracted from the food frequency questionnaires, where the participants were asked to indicate whether any special conditions influenced their diet. The question was phrased “Does any of the following alternatives influence your diet? You can select more than one alternative”. The alternatives included: “I am a vegetarian; I do not follow a Norwegian diet; I am allergic; I have a chronic disease; I have anorexia; I have bulimia; I am trying to lose weight; Diet with low glycemic index”. If a participant did not select any of the above-mentioned alternatives, it was interpreted as “no”. Therefore, there was no missing information on this question and DE was defined as participants that indicated AN, BN, or both. There was no questions of other types of DE available.

The variables included in the present study were extracted from the same questionnaire as the information about DE, except age at inclusion, which was extracted from the National Registry of Norway. Age was further categorized into three groups; 46–55, 56–65 and 66–76 years old, respectively. Body mass index (BMI) was calculated from self-reported weight and height, and used in a continuous form. The validity of self-reported height and weight in NOWAC has been found satisfactory [24]. Education was categorized into three groups based on years of completed education, corresponding to secondary school (<10 years), high school (10–12 years) and higher education (>12 years). Employment status was categorized into three groups; employment (full-time or part-time), retired and unemployed. Marital status was included as a dichotomous variable as having a partner (married or domestic relationship) or not. Physical activity was measured on an ordinal scale from 1–10, and further categorized into groups of low (1–4), moderate (5–6) and high (≥7) physical activity. The self-reported physical activity in NOWAC has been found to be valid [25]. Alcohol intake measured in gram per day was included as a dichotomous variable indicating higher or lower consumption than the median intake. Total energy intake (kcal/day) was calculated from the comprehensive food frequency questionnaire and used as a dichotomous variable similar to alcohol intake. The NOWAC food frequency questionnaire has been validated against repeated 24h dietary recalls and found valid [26]. Smoking status was assembled in the categories; never, former and current. Self-rated health was assembled in three categories; very good, good or poor/very poor. The participants were asked whether they were ever diagnosed with depression by a doctor and this information was included as a dichotomous variable (yes/no) in the regression analysis.

### Statistical analyses

Characteristics of women with DE were compared to women with no DE using independent t-test, Kruskal Wallis test and chi-square test, when appropriate. Multiple logistic regression analysis was used to identify the variables associated with DE and the strength of their relationships. The analyses were performed in accordance with the model building strategy for logistic regression described in Veierød et al [27]. Briefly, univariable analyses were used to explore the unadjusted associations between individual variables and DE as outcome. Variables with a significance level of p<0.25 were selected for inclusion in the multivariable analysis. Stepwise elimination of variables with p>0.05 was applied, and the full and the reduced models were compared using likelihood ratio tests. The percentage change of the regression coefficients between the full and reduced model was also assessed. If the regression coefficients changed by more than 20% after exclusion of a variable, that variable was re-entered into the model even if the likelihood ratio test suggested that it was not important for describing the odds of DE. The final model included only variables that were significantly associated with DE after mutual adjustments, or variables that confounded the effects of the other variables. Finally, we examined diagnostic plots of the residuals and tested the final model for overall goodness-of-fit using the Hosmer-Lemeshow test. Results are presented as crude and adjusted odds ratios (OR) including 95% confidence intervals (CI). Observations with any missing values on the included variables were excluded from the model. After cross-tabulations, we made an *a priori* decision not to test for interactions due to the lack of statistical power. All statistical analyses were conducted using the statistical software R version 3.4.3 and statistically significant results were defined as p-values <0.05.

## Results

Of the 90 592 women included in the present study, 253 reported DE by either selecting AN, BN or both in the questionnaires. This corresponds to an overall period prevalence of 0.28% (95% confidence interval 0.25–0.31%) during 2002–2005 among Norwegian women aged 46–76 years. The prevalence of DE in the oldest age group (≥ 66 years) was significantly higher (0.65% [95% confidence interval 0.60–0.70%]) than in the two younger age groups (Table 1).

**Table 1:** Total prevalence of disordered eating in 2002–2005 and stratified by age. The Norwegian Women and Cancer Study (n = 90 592).

Age-group (years)	Disordered eating % (n)	95% confidence interval
Total: 46–76	0.28 (253)	0.25–0.31
≤ 55	0.24 (111)	0.21–0.27
56–65	0.26 (92)	0.23–0.29
≥ 66	0.65 (50)	0.60–0.70

The 253 women with DE differed significantly (p <0.05) from the women without DE on several sociodemographic and lifestyle-related characteristics (Table 2). After mutual adjustments, DE was strongly associated with ever being diagnosed with depression (Odds Ratio [OR] 3.34 [95% confidence interval 2.53–4.41]), being unemployed (OR 1.78 [1.32–2.40]) and single (OR 1.66 [1.25–2.20]). In addition, women with DE were more likely to report a low energy intake (OR 1.41 [1.08–1.86]) and less likely to perform moderate physical activity (OR 0.67 [0.47–0.95]) than women without DE (Table 3). In the logistic regression analysis, 39 women with DE that had missing information on the investigated characteristics were excluded. Characteristics according to DE status in the sample with complete observations (n = 214, S1 Table) differed only slightly from the initial sample. Specifically, there was no significant difference in education level between women with and without DE in the sample with complete observations.

**Table 2:** Characteristics of women with and without disordered eating. The Norwegian Women and Cancer Study (n = 90 592), 2002–2005.

	Disordered eating n = 253, median (25th–75th percentile) or mean (SD) or %	No disordered eating n = 90 339, median (25th–75th percentile) or mean (SD) or %	p	Sample size
Age (years)	56 (51–63)	55 (51–59)	**<0.001**	90 592
BMI (kg/m^2^)	25.8 (5.9)	25.3 (4.1)	0.14	87 161
Education			**0.008**	85 739
Secondary school	30.0	22.7		20 539
High school	28.9	32.6		29 536
Higher education	33.2	39.4		35 664
Employment status			**<0.001**	90 592
Employed	39.5	60.9		55 161
Retired	19.4	10.0		9 120
Unemployed	41.1	29.0		26 311
Partner	63.6	78.2	**<0.001**	90 592
Physical activity			**<0.001**	81 310
Low	26.9	22.2		20 106
Moderate	23.3	37.1		33 596
High	34.4	30.5		27 608
Alcohol intake (g/day)	1.12 (0.00–3.84)	1.75 (0.40–5.25)	**<0.001**	83 479
Total energy intake (kcal/day)	1521 (1175–1870)	1630 (1334–1950)	**0.002**	90 592
Smoking status			0.12	88 095
Never	30.0	32.8		29 707
Former	36.8	40.4		36 551
Current	29.2	24.1		21 837
Self-rated health			**<0.001**	86 960
Very good	19.8	28.0		25 384
Good	55.3	60.3		54 589
Poor/very poor	18.6	7.7		6 987
Depression, diagnosed	42.7	18.5	**<0.001**	90 592

**Table 3:** Logistic regression estimates for disordered eating in The Norwegian Women and Cancer Study 2002–2005. Complete case analysis (n = 81 310, n case = 214). Reference group for the dependent variable was women with no disordered eating (n = 81 096).

	Beta coefficient	Standard error	Odds ratio	95% confidence interval	p
Age at inclusion					
45–55 years			1.00		
56–65 years	-0.071	0.15	0.93	0.69–1.25	0.64
66–76 years	0.579	0.32	1.78	0.94–3.34	0.07
Employment status					
Employed			1.00		
Retired	0.535	0.31	1.71	0.91–3.06	0.08
Unemployed	0.576	0.15	1.78	**1.32–2.40**	**<0.001**
Marital status					
Partner			1.00		
Single	0.509	0.15	1.66	**1.25–2.20**	**<0.001**
Physical activity					
Low			1.00		
Moderate	-0.404	0.18	0.67	**0.47–0.95**	**0.02**
High	0.263	0.17	1.30	0.94–1.81	0.11
Total energy intake					
High			1.00		
Low	0.346	0.14	1.41	**1.08–1.86**	**0.01**
Diagnosed depression					
No			1.00		
Yes	1.207	0.14	3.34	**2.53–4.41**	**<0.001**

## Discussion

In the present study of a representative sample of 90 592 women between 46 and 76 years of age from Norway, we estimated an overall period prevalence of 0.28 (95% confidence interval 0.25–0.31) % of DE during 2002–2005. This finding clearly supports the accumulating evidence that DE do occur also in women in mid-life and in older ages [7, 8, 28–32]. Along with previous studies [10], our finding that the oldest age group (66–76 years) had the highest prevalence of DE contradict the notion that DE and eating disorder symptoms decline throughout the life span [12, 14].

The prevalence rate of 0.28% of DE in 2002–2005 suggest that DE is not a frequent problem among Norwegian women above 45 years. As the definition of DE is highly variable in previous literature, comparison of prevalence rates across studies are difficult. The perhaps closest comparison with respect to sample size and population is the study by Eik-Nes et al. [13] who reported a prevalence of DE of 12% in 2006–2008 among close to 25 000 Norwegian women between 30 and 99 years from mid-Norway. The huge discrepancy in prevalence compared to our findings is noteworthy, but may at least partly be explained by how DE was measured. For obvious reasons lower prevalence figures may be obtained by alluding to AN or BN than by using self-report questionnaires where eating patterns are scored and interpreted as DE indicators without necessarily being perceived as deviant by the respondents.

Depression was the strongest correlate of DE in the present study (Table 3). Women reporting DE had more than three-fold higher odds of ever being diagnosed with depression. Being unemployed and single were further associated with increased odds of DE. These findings suggest that DE and clinically significant eating disorders share common correlates as comorbid mood disorders [18, 33, 34], and impairment in role functioning at home, work or in social life [2, 11] have been reported in previous population-based studies of eating disorders. This argument is however, challenged as the correlation between increased alcohol intake and eating disorders [17, 35] was not observed in the present study (Tables 2 and 3 and S1 Table). The relationship between DE and physical activity in our study were U-shaped, where both low and high physical activity was associated with DE. High physical activity levels have been associated with AN in previous studies [36, 37]. Apart from a few studies [38, 39] indicating that low physical activity may be associated with recovery from AN in younger females, the relationship between AN and low physical activity is rather unknown.

Our results showed that DE and BMI were unrelated, which contrast other studies, e.g. Ng et al., 2013 [9]. This could be explained by the fact that the NOWAC questionnaire did not include a question about BED, where overweight is prevalent. Additionally, over- and underestimations of body weight among women with AN and/or BN may have caused measurement errors as people in general, as well as those having BN, tend to underestimate their weight and overestimate their height [40], while people with AN may overestimate their weight [41, 42] as a reflection of their fear of over-weight. This fear may also lead to an avoidance of any questions about their weight, indicated by the fact that the amount of missing information on BMI was slightly higher in the DE group (5.9%) as compared to the no DE group (3.8%) in our study. Similar findings of no difference in body weight between women with and without eating disorders have been reported in previous population-based studies among women aged 15–50 years [12, 43]. Additional analyses comparing the AN and the BN groups could have been relevant. However, as AN and BN cases were identified from only one question and many respondents ticked off both the AN and the BN option, such analyses were not considered. In addition, we did not have statistical power to assess AN or BN in stratified analyses.

Important strengths of this study are the large and nationally representative sample of Norwegian middle-aged women, and the comprehensive information about each woman’s lifestyle, and sociodemographic characteristics. Moreover, considering the large variations in how DE is operationalized in the literature, DE was more precisely defined as self-reported AN and/or BN. As the general population is fairly well informed about the features of AN and BN in the community [44], this operationalization may prevent overestimated prevalence figures of DE by capturing trivial problems within a normal variation, and with negligible psychological significance. Previous studies have suggested that the prevalence of AN and BN have been relatively stable in recent years among subjects above 40 years of age [45]. A similar trend has been observed in younger women as well [45–47]. Thus, although our data was collected more than 10 years ago, the presented numbers are likely representative for the current situation among Norwegian middle-aged women. Our data can also serve as a benchmarking study for future studies of time-trends of DE among Norwegian women as the estimates are national representative. Moreover, the associations between DE and sociodemographic and lifestyle factors are not likely to be affected by the time of data collection.

The NOWAC study was not specifically designed for studying DE or its diagnostic counterparts. One may therefore have evaded the known underreport caused by people with DE or eating disorders in the population who do not sign up for a survey on DE [48] for reasons related to the (self)-stigmatization of suffering from DE and eating disorders [49–53]. On the other hand, the present study cannot inform about the behavioral and cognitive aspects of DE or the prevalence of AN or BN determined by diagnostic screening tools for eating disorders [54]. The failure to include questions about BED is unfortunate given the high prevalence of BED symptoms, particularly in the older age cohorts of the general population [21, 29, 30, 32, 55]. Thus, the prevalence figures reported in the present study may be regarded as conservative estimates of the true population prevalence. In addition, the detection of possible correlates of DE was hampered by the low number (n = 214) of self-reported DE in the final regression model, and the cross-sectional design limited our ability to assess causality.

To conclude, the present study confirms previous smaller scaled studies in showing that DE do occur in mid-life and older age women. We have also identified factors associated with DE in this population. Our results contribute to address a somewhat under-communicated community health problem that needs attention in terms of age-specific treatment and prevention.

## Supporting information

S1 Table Characteristics of women with and without disordered eating included in the logistic regression analysis. The Norwegian Women and Cancer Study (n = 81 310), 2002–2005.
(DOCX)
