# Supplementary material for: Prevalence and correlates of self-reported disordered eating: A cross-sectional study among 90 592 middle-aged Norwegian women
Source: PLoS One. 2019 Jan 23;14(1):e0211056. doi: 10.1371/journal.pone.0211056 (PMC6343912; doi:10.1371/journal.pone.0211056)
Supplement: S1 Table — The Norwegian Women and Cancer Study (n = 81 310), 2002–2005. (DOCX) [file pone.0211056.s001.docx]

**Supporting Information for the manuscript entitled:**

# **Prevalence and correlates of self-reported disordered eating: a cross-sectional study among 90 592 middle-aged Norwegian women**

Marie Sigstad^1^, Jan H. Rosenvinge^2^, Guri Skeie ^1^, Charlotta Rylander^1^

^1^Department of Community Medicine, UiT The Arctic University of Norway, Tromsø, Norway

^2^Department of Psychology, UiT The Arctic University of Norway, Tromsø, Norway

S1 Table. Characteristics of women with and without disordered eating included in the logistic regression analysis. The Norwegian Women and Cancer Study (n = 81 310), 2002-2005.

|  |  | Disordered eating,  n=214, median  (25th - 75th percentile) or mean (SD) or % | No disordered eating, n=  81 096, median  (25th - 75th percentile) or mean (SD) or % | p |
| --- | --- | --- | --- | --- |
| Age (years) | | 55 (51-60) | 55 (51-59) | **0.045** |
| BMI (kg/m^2^) | | 25.8 (6.1) | 25.2 (4.1) | 0.19 |
| Education | |  |  | 0.20 |
|  | Secondary school | 24.8 | 20.3 |  |
|  | High school | 30.8 | 33.4 |  |
|  | Higher education | 37.9 | 41.7 |  |
| Employment status | |  |  | **<0.001** |
|  | Employed | 43.0 | 63.1 |  |
|  | Retired | 15.0 | 8.0 |  |
|  | Unemployed | 42.0 | 28.9 |  |
| Partner | | 64.0 | 79.0 | **<0.001** |
| Physical activity | |  |  | **<0.001** |
|  | Low | 31.8 | 24.7 |  |
|  | Moderate | 27.6 | 41.4 |  |
|  | High | 40.7 | 33.9 |  |
| Alcohol intake (g/day) | | 1.47 (0.00-4.47) | 1.97 (0.46-5.47) | **0.001** |
| Total calorie intake (kcal/day) | | 1535 (1243-1900) | 1648 (1359-1964) | **0.004** |
| Smoking status | |  |  | 0.10 |
|  | Never | 28.5 | 32.6 |  |
|  | Former | 38.3 | 40.8 |  |
|  | Current | 30.4 | 24.4 |  |
| Self-rated health | |  |  | **<0.001** |
|  | Very good | 21.0 | 29.1 |  |
|  | Good | 55.6 | 67.1 |  |
|  | Poor/very poor | 18.7 | 7.5 |  |
| Depression, diagnosed | | 46.7 | 19.0 | **<0.001** |
